# Characteristics of children born to mothers with an anorectal malformation

**DOI:** 10.1007/s00383-025-06278-2

**Published:** 2025-12-27

**Authors:** Joshua Gertler, Anna Gunnarsdóttir, Jenny Oddsberg, Anna Svenningsson, Lisa Örtqvist, Anna Löf Granström, Tomas Wester

**Affiliations:** 1 Department of Pediatric Surgery, Karolinska University Hospital, C11:33, Solna, Stockholm, 17176 Sweden; 2 Department of Women’s and Children’s Health, Karolinska Institutet, Stockholm, Sweden

**Keywords:** Anorectal malformation, Parity, Offspring, Inheritance, Delivery mode, Pregnancy, SGA, Congenital malformations

## Abstract

**Purpose:**

To describe characteristics of offspring born to mothers having an anorectal malformation (ARM).

**Methods:**

This was a national population-based cohort study. The exposed group was the offspring of females with an ARM diagnosis (ICD-codes) in the Swedish National Patient Register 1964–2008. The unexposed group was the offspring of mothers without ARM. Five age-matched mothers without ARM were randomly identified for each mother with ARM by Statistics Sweden. Females with chromosomal aberrations were excluded prior to analysis. Outcomes of the mothers were retrieved from the National Patient Register (1964–2021) and Medical Birth Register (1973–2021). Outcomes of the offspring were based on data from Medical Birth Register. Using descriptive statistics, the children of mothers with an ARM were compared to children of mothers not having an ARM. The ethical review authorities approved the study.

**Results:**

The mothers included 464 females with ARM and 2313 females without ARM. Of the 464 females with ARM, 132 (28.4%) gave birth to at least one child compared with 895 (38.7%) females without ARM (*p* < 0.001). The 132 first-born children of mothers with ARM were born at an 8-day younger median gestational age (272 days) when compared to children to mothers without ARM (280 days) (*p* < 0.001). The infants to mothers with ARM were more commonly small for gestational age (*n* = 10, 7.6%) than infants to mothers without ARM (*n* = 28, 3.1%) (*p* = 0.012). Initial results also showed that the first offspring to ARM mothers were statistically lighter and shorter than the offspring to mothers without ARM (*p* < 0.001), however, after adjusting for gestational age in a linear regression model, this was no longer the case (*p* = 0.398 and *p* = 0.255, respectively). Three first-born offspring in the group of mothers with an ARM, whereas no offspring from mothers without ARM had an ARM.

**Conclusion:**

The first-born child of mothers with ARM are more commonly small for gestational age than the first-born of mothers without ARM. The offspring to mothers with ARM more commonly had an ARM.

*Level of evidence* III.

## Highlights

- What is currently known about this topic?

Little is known regarding characteristics of the offspring to mothers with an anorectal malformation (ARM).

- What new information is contained in this article?

The first-born of mothers with ARM are more commonly SGA than the first-born of mothers without ARM. A higher prevalence of ARM in the neonates to mothers with ARM suggests an autosomal inheritance pattern of ARM in certain cases.

## Introduction

Data on reproductive outcomes of women with anorectal malformations (ARM) is limited. This includes characteristics of the offspring to women with ARM. Müllerian duct anomalies, which are more common in females with ARM [1, 2], have been associated with preterm delivery of the offspring and short-term neonatal complications [3]. Furthermore, the frequency of neonatal adverse events such as low 5-min APGAR score, the need of NICU admission or intubation, and sepsis were significantly elevated in offspring to women with a Müllerian duct anomaly [3]. In this study, we aim to characterize the offspring to mothers with ARM.

Although the occurrence of ARM is usually sporadic and the etiology of ARM remains widely unknown, there have been suggestions of an autosomal inheritance pattern in at least a subset of ARM families [4]. Two other studies have shown that an individual with ARM have a sibling or other relative with ARM in roughly 1-1.4% [5, 6]. In the study by Dworschak et al. an inheritance risk of 62% from parent to offspring was described [6, 7]. In the present study, the aim was to characterize the offspring to women with ARM.

## Methods

### Study design

This was a national population-based cohort study.

### Study setting

The study comprised national Swedish register data. It focused on the offspring of females with ARM diagnosed in Sweden between 1964 and 2008.

### Participants

The exposed group was the offspring of females with an ARM diagnosis (ICD-codes) in the Swedish National Patient Register from 1964 to 2008. The unexposed group was the offspring of mothers without ARM. The diagnosis of ARM in the mothers was based on International Classification of Diseases (ICD) codes as a primary diagnosis in the National Patient Register (ICD-7, 756.10 or 756.11; ICD-8, any of 751,20 to 751,29 or 751,52; ICD-9, 751 C or 751 F; and ICD-10, any of Q42.0 to Q43.5 or Q43.7). Only females admitted to one of the pediatric surgery centers in Sweden that performed ARM repair at the time of inclusion were included. To be included, an age limit of < 15 years at time of diagnosis was set to minimize misclassification bias. Five age-matched females without ARM were randomly selected and provided by Statistics Sweden for whom the same data variables were then obtained through the National Patient Register and Medical Birth Register. Females with chromosomal aberrations, based on ICD codes, were excluded from the analysis. The final study subjects were the offspring to both the mothers with or without ARM. The outcomes of the offspring were based on data from the Medical Birth Register. The STROBE guidelines for reporting of observational studies were used [8]. In 2024, Sweden had a population of roughly 10.6 million persons [9].

### Data sources and variables

#### Registers

The National Board of Health and Welfare and Statistics Sweden administer the registers that were used in this study. All Swedish nationals have a unique personal identification number through which data in the registers can be linked. The National Patient Register, established in 1964, compiles patient data, amongst other variables, regarding diagnosis from both in and out-patient specialist care. The study’s inclusion time spans four different ICD versions (ICD-7 to ICD-10) which was taken into consideration when coding for inclusion and exclusion. In 2011, a validation study was done on the Swedish National Patient Register showing 85–95% diagnosis validity [10]. Further, we gained data from the National Medical Birth Register, started in 1973, which compiles data regarding all pregnancies resulting in childbirths.

#### Variables

The offspring outcomes included delivery mode, sex, APGAR at 5 min (APGAR-5), birth size (small for gestational age, SGA or large for gestational age, LGA), gestational age, birth length and weight and the presence of congenital malformations. All variables related to the offspring were retrieved from the Medical Birth Register. End of follow-up was set to the 31st of December 2021.

### Statistical methods

The cohort included all newborns to mothers who met the inclusion criteria. Categorical data are presented as frequencies analyzed using Fisher’s Exact test. Numeric variables were analyzed using the Mann–Whitney U test for non-normally distributed data. These variables are presented as medians and interquartile ranges (IQR). Statistical significance was set at *p* < 0.05. A multiple linear regression model was applied to compare birthweight and length of offspring from the exposed and unexposed mothers. The model adjusted for factors known to influence birthweight including mode of delivery and gestational age.

### Ethical considerations

The study was approved by the Swedish Ethical Review Authorities (Dnr 2022/00983-01).

## Results

### Patient characteristics

**Fig. 1:**
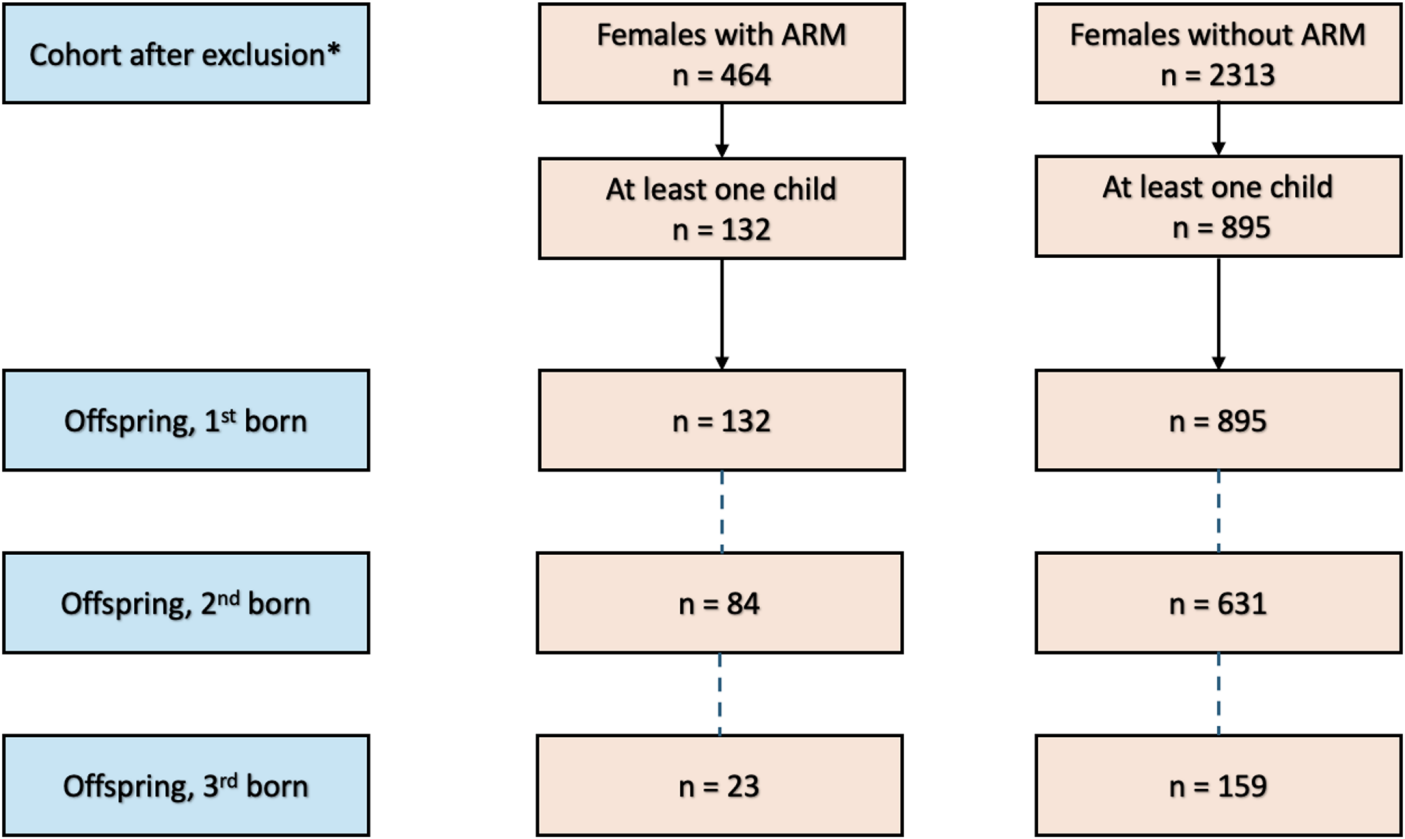
The participants’ inclusion process and number of offspring. *Exclusion through chromosomal aberrations, age at inclusion < 15, incompatible treatment center or non-primary diagnosis. ARM, Anorectal Malformation

The inclusion process and number of offspring are summarized in Fig. 1. Prior to exclusion there were 934 female persons with ARM identified and 4670 age-matched unexposed controls.

The mothers with and without ARM were comparable in the prevalence of gestational diabetes and preeclampsia, smoking habits, their age at primiparity and their highest attained educational level (unpublished data).

### Offspring characteristics

Characteristics of the first-born offspring are shown in Table 1. The first-born children to mothers with ARM are born at a lower gestational age (38 + 6 vs. 40 + 0 weeks) and were more commonly SGA, as well as being lighter and shorter than their counterparts in the unexposed group. When adjusted for gestational age using a linear regression model, birth weight and length no longer differed between the groups (*p* = 0.398 and *p* = 0.255, respectively). There was no significant difference between the exposed and unexposed group in terms of APGAR-5, or sex or overall proportion having a congenital malformation.

**Table 1:** First-born offspring characteristics at birth for 132 women with ARM and 895 controls

	Level	Exposed *n* = 132	Unexposed *n* = 895	*p*-value
Delivery mode, n (%)	Vaginal	53 (40.2)	751 (83.9)	< 0.001
	Caesarian	79 (59.8)	144 (16.1)	
APGAR-5, n (%)	7–10 p	130 (98.5)	868 (97.0)	0.891
Gestational age, weeks + days, median [IQR]		38 + 6 [38 + 0, 39 + 6]	40 + 0 [39 + 0, 41 + 0]	< 0.001
Congenital Malformation*, n (%)		8 (6.1)	35 (3.9)	0.245
ARM, n (%)		3 (2.3)	0 (0.0)	< 0.001
Sex, n (%)	Male	59 (44.7)	472 (52.7)	0.093
	Female	73 (55.3)	423 (47.3)	
SGA, n (%)		10 (7.6)	28 (3.1)	0.012
LGA, n (%)		3 (2.3)	19 (2.1)	0.263
Birth weight, g, median [IQR]		3243 [2815, 3640]	3473 [3120, 3820]	< 0.001
Birth length, cm, median [IQR]		49 [48,51]	50 [49, 52]	< 0.001

Congenital Malformation* ICD10: all Q diagnoses; ICD-9: 740–759. Abbreviation: ARM, anorectal Malformation; SGA, small for gestational Age; LGA, large for gestational Age. ** missing data between 0.0 and 1.6% of cases for listed variables

Characteristics of second and third-born children are summarized in Table 2. For the second-born children, offspring to ARM mothers were no longer significantly SGA compared to their controls, yet they were still shorter and lighter at birth (*p* < 0.001, not adjusted for mediators). For the third-born, none of these three variables differed between the groups (not adjusted for mediators), however, there was a significant difference in congenital malformation prevalence (17.4% if exposed and 2.5% if unexposed, *p* = 0.01). Looking at the presence of an ARM in first-born children, those born to exposed mothers had an ARM in 2.3% of cases.

**Table 2:** Unadjusted birthweight, length and presence of SGA in second and third-born offspring to exposed vs. unexposed mothers

2nd Born	Exposed *n* = 84	Unexposed *n* = 631	*p*-value
SGA, n (%)	2 (2.4)	5 (0.8)	0.085
Congenital Malformation*, n (%)	4 (4.8)	19 (3.0)	0.33
Birth weight, g, median [IQR]	3357 [2990, 3674]	3645 [3300, 3980]	< 0.001
Birth length, cm, median [IQR]	49.5 [48,51]	51 [49, 52]	< 0.001

3rd Born	Exposed n = 23	Unexposed *n* = 159	p-value
SGA, n (%)	1 (4.3)	3 (1.9)	0.49
Congenital Malformation*, n (%)	4 (17.4)	4 (2.5)	0.01
Birth weight, g, median [IQR]	3485 [3120, 3868]	3575 [3225, 3856]	0.64
Birth length, cm, median [IQR]	50 [49.5,51]	51 [49, 52]	0.67

Abbreviation: SGA, small for gestational age

## Discussion

### Key findings

To our knowledge, no previously published data exists describing SGA for offsprings of ARM patients. The first-born offsprings were more commonly SGA if their mother had an ARM compared to first-borns whose mother did not have an ARM. Further we found that offspring to mothers with ARM themselves more commonly have an ARM.

### Interpretation

A literature review including offspring outcomes was performed in 2023 by Bischoff et al., yet no concrete results were found [11]. Here, we found no significant difference in the first-born offspring’s sex, or APGAR at 5 min. We did, however, find that the first-borns to ARM mothers compared with those to unexposed mothers were more commonly SGA. A previous study has described neonates with ARM to be more frequently SGA [12], but this finding in the offspring to mothers with ARM have not previously been described. Unadjusted birthweight and length of first-born children differed between the groups, but this difference disappeared after adjustment using a multiple linear regression model. The unadjusted weight and length variable differences were, at least in-part, explained by our exposed subjects being delivered earlier. The lower gestational age can in turn partially be explained by the predominant cesarian section delivery mode found in the group of ARM mothers. These noteworthy mediators can, however, not be used to explain why SGA was more common in offspring to mothers with ARM. Hypothetically, Müllerian duct anomalies, being more common in ARM mothers [1, 2], could be an influential factor on the SGA variable, yet further studies are needed.

Furthermore, we found that three first-born offspring to mothers with ARM had an ARM diagnosis whereas no first-borns had an ARM in the larger group of offspring to mothers without ARM. These findings are in par with a study from 2017, where an autosomal dominant inheritance pattern for ARM was suggested. The study in question showed a 1500- fold increased recurrence risk of ARM if either parent had an isolated ARM, yet the number of parents and offspring included were very low [6]. This is contrasted by Falcone’s findings of a 90-fold (3%) increased risk for recurrence for offspring to a parent with a perineal or vestibular fistula [5]. As no first-born offspring to unexposed mothers had ARM in our cohort, it is not possible to determine the relative recurrence risk. Kundal et al. described two sets of male monozygotic twins with isolated ARM [13] further reinforcing the idea of an in-part autosomal inheritance pattern. In their review of monozygotic twins, nine cases had been identified up to the year of 2022.

### Limitations

A first limitation lies within the employed registers themselves which are not 100% accurate yet the National Patient Register has been deemed to have 85–95% diagnosis validity [10]. Whilst register studies cannot infer causality, they can describe associations. Another limitation rises from the nature of ICD-coding that did not allow for stratification per ARM-subtype which, as ARM has a heterogenous disease phenotype, limits the possibility of conclusions made per subtype. Further, the variable describing presence of congenital malformations is limited in the Medical Birth Register as it only covers clinically observed perinatal diagnoses in the children. This surely explains the low prevalence reported for congenital malformations in the children to exposed mothers. The small sample size makes interpretation uncertain and there is a risk for type 2 errors.

## Conclusion

First-born offspring to mothers with ARM are to a larger extent SGA when compared to offspring to mothers without ARM. The offspring to mothers with ARM more commonly had an ARM compared with offspring to mothers without ARM. Besides continued genetic research in the field, further efforts are needed to standardize obstetrical care of pregnant females with ARM.

## Data Availability

No datasets were generated or analysed during the current study.

## Abbreviations

APGAR: Appearance-pulse-grimace-activity-respiration
ARM: Anorectal malformation
ICD: International classification of diseases
IQR: Interquartile range
LGA: Large for gestational age
SGA: Small for gestational age
SD: Standard deviation

## Declarations

### Declarations of generative AI and AI-assisted technologies in the writing process

Artificial Intelligence (AI) and AI-assisted technologies were not used in the preparation of this manuscript.
